# Ultrasensitive On-Field Luminescence Detection Using a Low-Cost Silicon Photomultiplier Device

**DOI:** 10.1021/acs.analchem.1c00899

**Published:** 2021-05-11

**Authors:** Maria Maddalena Calabretta, Laura Montali, Antonia Lopreside, Fabio Fragapane, Francesco Iacoangeli, Aldo Roda, Valerio Bocci, Marcello D’Elia, Elisa Michelini

**Affiliations:** † Department of Chemistry “Giacomo Ciamician”, University of Bologna, 40126 Bologna, Italy; ‡ Center for Applied Biomedical Research (CRBA), University of Bologna, 40126 Bologna, Italy; § Gabinetto Regionale di Polizia Scientifica per l’Emilia-Romagna, 40123, Bologna, Italy; ⊥ INFN, Istituto Nazionale di Fisica Nucleare Sezione di Roma, 00185 Rome, Italy; ¶ INBB, Istituto Nazionale di Biostrutture e Biosistemi, 00136 Rome, Italy; □ Health Sciences and Technologies-Interdepartmental Center for Industrial Research (HST-ICIR), University of Bologna, 40126 Bologna, Italy

## Abstract

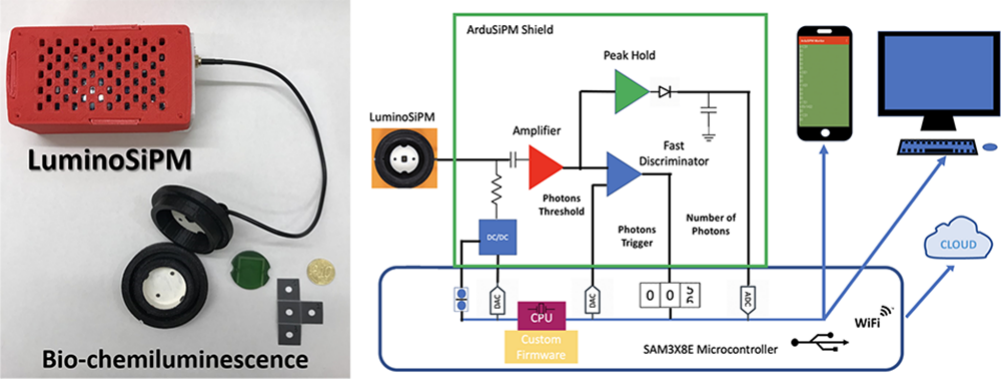

The availability of portable analytical devices for on-site monitoring and rapid detection of analytes of forensic, environmental, and clinical interest is vital. We report the development of a portable device for the detection of biochemiluminescence relying on silicon photomultiplier (SiPM) technology, called LuminoSiPM, which includes a 3D printed sample holder that can be adapted for both liquid samples and paper-based biosensing. We performed a comparison of analytical performance in terms of detectability with a benchtop luminometer, a portable cooled charge-coupled device (CCD sensor), and smartphone-integrated complementary metal oxide semiconductor (CMOS) sensors. As model systems, we used two luciferase/luciferin systems emitting at different wavelengths using purified protein solutions: the green-emitting *P. pyralis* mutant Ppy-GR-TS (λ_max_ 550 nm) and the blue-emitting NanoLuc (λ_max_ 460 nm). A limit of detection of 9 femtomoles was obtained for NanoLuc luciferase, about 2 and 3 orders of magnitude lower than that obtained with the portable CCD camera and with the smartphone, respectively. A proof-of-principle forensic application of LuminoSiPM is provided, exploiting an origami chemiluminescent paper-based sensor for acetylcholinesterase inhibitors, showing high potential for this portable low-cost device for on-site applications with adequate sensitivity for detecting low light intensities in critical fields.

In recent years, with the increasing necessity to perform rapid, cheap, and sensitive on-site detection of different analytes such as environmental pollutants, pathogens, and food contaminants, researchers have sought to develop new bioanalytical tools and new portable detectors. This urgency stems from different areas spanning diagnostics to food safety and quality control, environmental monitoring, and forensic fields.^1−3^ A number of portable devices and biosensors have been reported; however, most of them are currently at the prototype stage, and very few reached the market, with glucose sensors and adenosine triphosphate (ATP) detection devices dominating the landscape.^4−8^ Among main bottlenecks that hamper commercialization and use of these devices for real life applications, the relatively high cost, inadequate sensitivity when applied to real samples, and difficulty of interfacing with electronics are surely some of the main factors.^9^

Different optical detection techniques have been implemented in portable analytical devices, including fluorescence, biochemiluminescence (BL-CL), and colorimetric detection.^10−13^ BL-CL presents significant advantages especially in terms of sensitivity, low cost, and ease of integration with miniaturized systems.^14,15^ Different portable light detectors have been exploited to detect BL-CL signals, including photomultipliers (PMT), charge-coupled devices (CCDs), complementary metal oxide semiconductor (CMOS) sensors, and smartphone-integrated CMOS.^16−20^ Due to their high efficiency, PMTs are the most used detectors. However, the PMT technology has relevant drawbacks, such as intrinsic complexity and fragility, high energy consumption and requirement of high voltages.^21^ Portable CCD and CMOS showed feasible alternatives for both bioluminescence (BL) and chemiluminescence (CL). In the last years smartphone-integrated sensors (CMOS) showed suitable to replace other portable detectors, including CCDs and CMOS.^22−24^

Silicon photomultipliers (SiPMs) have also been proposed as sensitive light detectors for BL.^25,26^ SiPMs are arrays of avalanche photodetectors working in Geiger mode (GM-APDs), each one having integrated passive-quenching resistor and connected in parallel.^27^ Due to their high quantum efficiency, low energy and bias voltage requirements, and rapid fast response time (in nanosecond time scale), SiPMs have attracted significant interest for various applications, providing in some cases better performance than smartphones.^25−28^ Despite jeopardized efforts, there is no clear evidence about the selection of the most suitable light detector for implementation into portable devices and scaled-up deployments. This choice in fact should consider not only the sensitivity but also the cost, ruggedness, and ease of integration.

In the present study we evaluated the implementation of BL-CL reactions in a SiPM device and performed a side-by-side comparison of its performance with other portable light detectors that showed very good performance in previous works. We selected as model analytes a green emitting *P. pyralis* firefly luciferase mutant Ppy-GR-TS (λ_max_ 550 nm)^29^ and the blue-emitting NanoLuc luciferase (λ_max_ 460 nm).^30^

The SiPM device outperformed other portable detectors, providing adequate sensitivity for detecting low light intensities and making it suitable for on-site and forensic applications. As a proof-of-concept application, a CL origami sensing paper for the rapid detection of organophosphorus (OP) pesticides was implemented in the device to assess its analytical performance.

## Experimental Section

A full list of reagents and a detailed description of methods can be found in the Supporting Information.

### LuminoSiPM Fabrication

**Figure 1:**
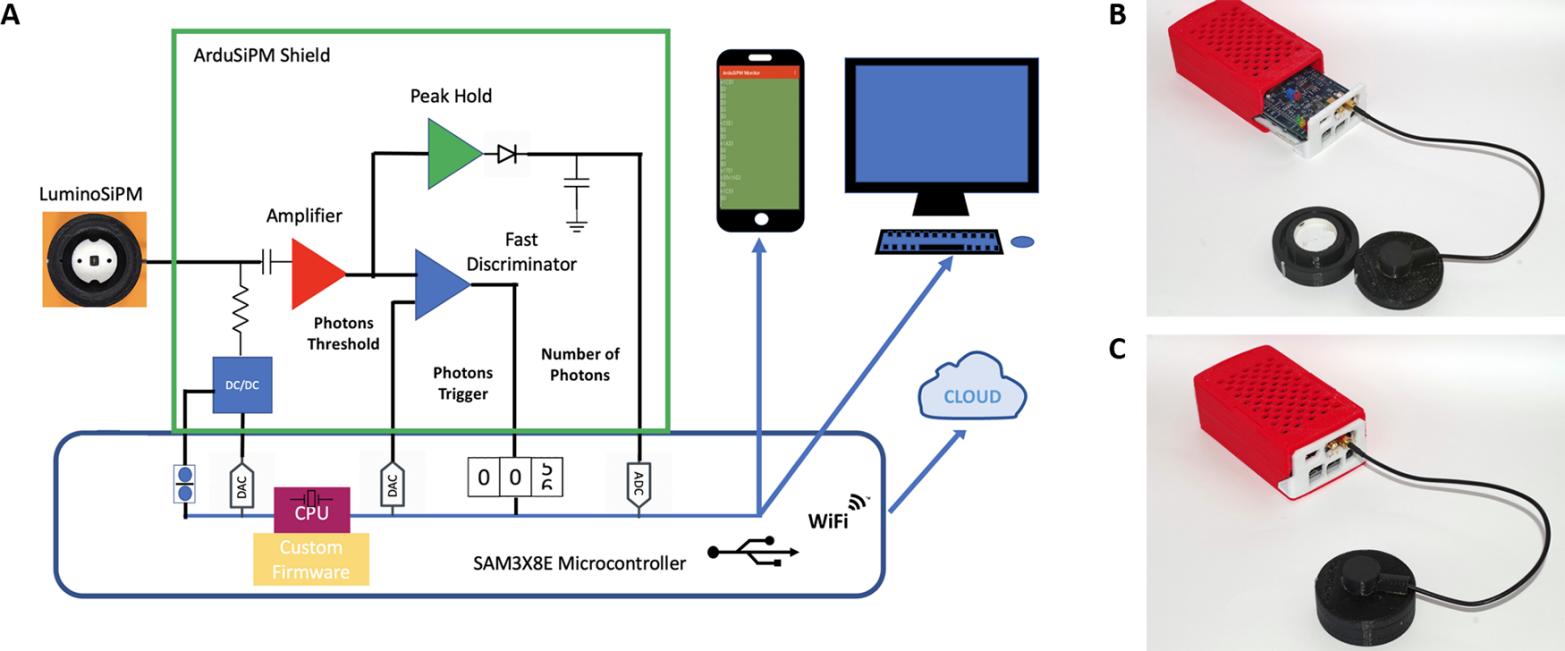
(A) ArduSiPM block diagram composed by the dark box with SiPM and temperature sensors, an internal digital controlled DC-DC converter as voltage supply, a voltage amplifier, a fast discriminator with programmable threshold, a peak hold circuit for pulse amplitude, LEDs for monitoring; all outputs from analog circuit and digital controls are connected to the Arduino DUE board. (B) Ardu-SiPM (red-white box) connected to LuminoSiPM dark box (black cell) ready for sample addition and (C) closed for acquisition.

The LuminoSiPM device is composed of a SiPM sensor (Hamamatsu MPPC 13360-1325CS) read by means of the ArduSiPM system^31,32^ (Supporting Information) integrated in a portable case and designed with FreeCAD software. It is able to accommodate in its inner cavity different types of disposable sample holders for hosting biospecific reactions (Figure 1 and Figure S1).

### LuminoSiPM Signal Acquisition and Data Treatment

The ArduSiPM is an all-in-one detector that processes the SiPM signal independently using customized firmware and shows all measurements about the events directly throughout a serial interface. For an improved treatment of BL-CL signal acquisition, a specific Microsoft Windows software was used.^33^ In all measurements, the sensor’s signal was acquired for 5 min with data sampling cycles of 1 s. A total of 300 numerical acquisitions were recorded in the form of counts per second (cps) for any single experiment and stored in CSV format.

#### Evaluation of LuminoSipM Analytical Performance

To investigate the suitability of LuminoSipM to detect low light intensities of BL reactions, PpyGR-TS and NanoLuc luciferases were purified (Supporting Information).^29^ A 5 μL volume of purified protein solution (concentration range from 1.0 to 1.0 × 10^–6^ mg/mL) was dispensed in the sample holder cartridge shown in Figure S1, and BL acquisition was performed after addition of 10 μL of BL substrate, i.e., BrightGlo and NanoGlo substrates (Promega) for PpyGR-TS and NanoLuc, respectively. Acquisitions with LuminoSipM were performed for 5 min with data sampling cycles of 1 s. Comparative studies were performed with different portable light detectors, including a Oneplus 6 smartphone (Oneplus, Shenzhen, China) equipped with an integrated dual camera (16 MP Sony Exmor IMX 519 sensor and F1.7 aperture +20 MP Sony Exmor IMX 376 K sensor and F1.7 aperture) and a portable CCD camera (ATIK 383L+ mono cromo CCD) equipped with a high-resolution monochrome CCD sensor (Kodak KAF 8300, sensor size 17.96 × 13.52 mm). The limit of detection (LOD) was calculated as the blank plus three times the standard deviation. All details of BL measurements are described in the Supporting Information.

#### LuminoSiPM-Based Detection of Acetylcholinesterase (AchE) Inhibitors with a Chemiluminescent Origami Sensing Paper: Analytical Procedure

An origami sensing paper previously described^23^ was modified to fit the LuminoSiPM and the analytical procedure adapted. The sensing paper consisted of four circular hydrophilic “wells” with a diameter of 5 mm, surrounded by hydrophobic areas obtained by wax printing. The enzymes, i.e, AChE, choline oxidase (ChOx), and horseradish peroxidase (HRP), were loaded in the wells of the sensing paper via physical adsorption with cellulose matrix. The full description of the analytical procedure is provided in the Supporting Information.

## Results and Discussion

### LuminoSiPM Device Fabrication

A compact and portable device, called LuminoSiPM, was developed with the aim of exploiting SiPM technology to measure the photons produced by BL-CL systems as an alternative to more explored portable light detectors. To provide an all-in-one device, a removable plastic box was 3D printed to encase the ArduSiPM and enable easy inspections on functionality and operative conditions (Figure 1B, C). The LuminoSiPM cell was connected to the signal inlet of ArduSiPM (SMA connector); data acquired were transferred in real-time to a PC via the ArduSiPM USB port for data storing and software treatment. Electric power was supplied by the PC USB port (5 V, current max 0.5 A) (Figure 1A).

In SiPM sensors, increasing the operating voltage improves photon detection but produces a significant increase in noise components in terms of dark count and crosstalk. Nevertheless, the Hamamatsu SiPMs S13360 series, as declared by the company, has a reduced noise increase when operating voltage is increased. This is highly advantageous for detecting low-light signals similar to those generated by BL-CL reactions because the signal gain can reach the 1 × 10^6^ limit in terms of photoelectrons’ amplification, a value comparable with PMT’s response. Improvement in material and design technology permitted reduction of dark counts down the Mcps threshold (range from 70 to 210 Kcps at 25 °C). As concerns the LuminoSiPM data output, they are available as ASCII format through an RS232 serial interface with maximum speed of 115 200 Baud. Therefore, data can be easily acquired using high integrated computer platforms such as smartphone, Raspberry Pi, or simple microcontroller devices like M5Stack. These properties support the suitability of LuminoSiPM as a portable photometric instrument for operation in the field, even in uncontrolled settings.

### Optimization of SiPM Sensor Driving Parameters

The original use of ArduSiPM was coupled with a scintillator in a radiation detector. In this application, the threshold usually adopted is in the range of five photoelectrons. This value provides a dark count near to zero cps suitable for rare yet intense signals produced when an ionizing particle hits the scintillator and yields a few-nanosecond burst of photons. Conversely, for BL detection, reactions lead to a continuous emission of photons. With a threshold of five photons, the detector does not count events when there is an emission of one, two, three, or four photons in a microsecond-scale window. Therefore, considering the typical photon generation of BL reactions, a threshold of about 2–3 photoelectrons was selected. With a two-photoelectron threshold, a composed dark count of a few hundreds of cps was obtained. Although this threshold did not allow the detector to reach the physical limit of one photoelectron, it was still suitable to measure very low light intensities as BL-CL signals with a good signal-to-noise ratio (SNR) even at room temperature. A series of measurements was performed to identify the optimal conditions to achieve the highest SNR. After the preamplification stage of the SiPM signal, a photoelectron corresponded to a voltage of about 2.0 mV. Dark noise optimization was achieved by varying the threshold with 0.1 mV increments in the range of 3.39–4.57 mV. Measurement sessions were performed at 25 ± 1 °C (A series) and 27 ± 1 °C (B series) with the sensor blinded in a time window of 5 min to avoid the contribution of temperature drift. Besides an expected temperature dependence, a significant noise increase was observed with variation of a few millivolts, highlighting the threshold mechanism of the process (Figure S2). The optimal threshold was in the range of 3.55–3.60 mV, corresponding to two photons hitting the sensor with an integrated noise between 2300 and 1900 Hz (cps). Below the 3.0 mV threshold (1.5 photoelectron equivalent), the dark count increased rapidly up to tens of thousands of cps. A lower threshold value produced an abrupt increase both in the absolute number of counts and in the occurrence of multiple pulses of noise. The bias voltage was set at 3.60 mV to maximize sensor sensitivity while keeping acceptable dark noise figures (approximately 2000 spurious counts in 5 min acquisition at 25 °C).

### Evaluation of LuminoSiPM Performance and Comparison with Other Light Detectors

**Figure 2:**
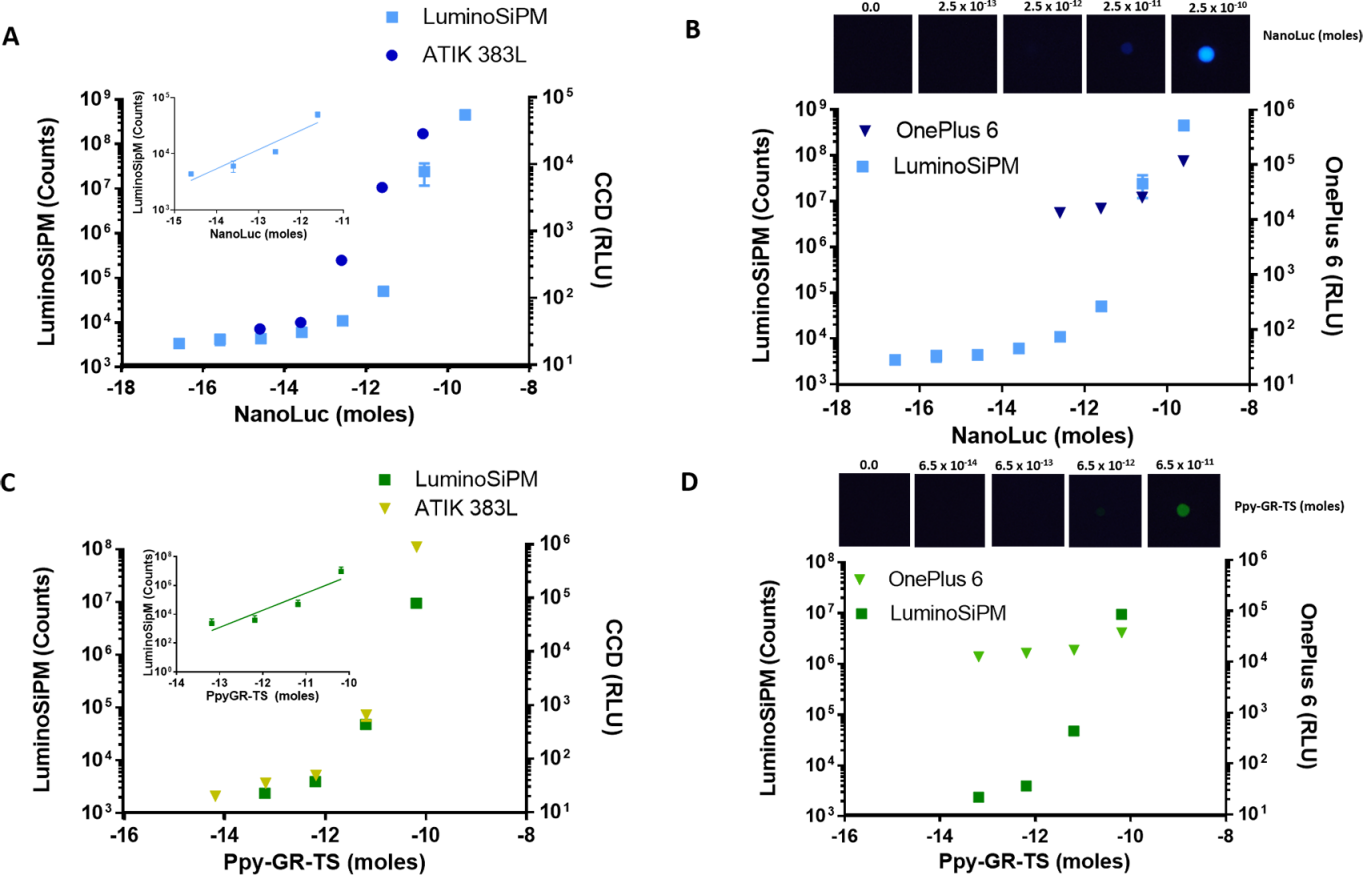
Calibration curves for Nanoluc and PpyGR-TS obtained with the LuminoSiPM, Oneplus 6, and CCD camera. (A) Calibration curve for NanoLuc and (C) PpyGR-TS obtained with the LuminoSipM and CCD camera. (B) Calibration curve for NanoLuc and (D) PpyGR-TS obtained with the LuminoSipM and OnePlus 6 smartphone. Representative images obtained with the Oneplus6 are also shown.

To assess the feasibility of employing LuminoSiPM as a light detector for low light intensities, we first compared the sensitivity of the system in detecting BL emitted by purified luciferase solutions. A blue emitting luciferase (NanoLuc) and a green emitting luciferase variant of the *P. pyralis* luciferase (Ppy-GR-TS) having λ max at 460 (half bandwidth 70 nm) and 548 nm (half bandwidth 66 nm), respectively, were selected as models of BL emission (Figure S3). We obtained calibration curves for the two luciferases (in the range 1 pg μL^–1^ to 1 μg μL^–1^) employing either d-luciferin/ATP/Mg^2+^ or furimazine substrates, respectively. A LOD of 4.2 × 10^–8^ M, corresponding to 2.1 × 10^–13^ moles, and a LOD of 1.7 × 10^–10^ M, corresponding to 8.7 × 10^–16^ moles, were obtained for Ppy-GR-TS luciferase and NanoLuc luciferase, respectively. We compared the LODs and linear range with those obtained with a benchtop luminometer, a portable CCD camera (ATIK 383L+), and CMOS-smartphone integrated sensors (OnePlus6). Both the selected CCD camera and the CMOS-smartphone sensors previously demonstrated their suitability as portable light sensors for BL and CL assays.^23,24^ The OnePlus smartphone-integrated CMOS was reported to be the best performing smartphone-integrated sensor by Kim et al., who performed a comparison with five different types of smartphones and reported that best results were achieved by OnePlus One.^34^Figure 2 shows concentration–response curves obtained with the three portable light detectors (ATIK 383L+, OnePlus 6 smartphone, and SiPM sensor). Calibration curves obtained with a benchtop luminometer, used as reference instrumentation, provided an LOD of 4.3 × 10^–10^ M (2.1 × 10^–15^ moles) and an LOD of 6.4 × 10^–11^ M (3.2 × 10^–16^ moles) for PpyGR-TS and NanoLuc luciferases, respectively (Figure S4).

The calculated LODs for Ppy-GR-TS and NanoLuc were one and two orders of magnitude lower, respectively, than those obtained with the CCD ATIK 383L. This unexpected result was only partially explained by the high sensitivity of the SiPM sensor at the wavelengths of the NanoLuc emission spectrum (quantum efficiency of 25% at 450 nm)^35^ because reported quantum efficiency of the KAF-8300 sensor of the ATIK 383L+ camera shows a similar behavior, reaching about 45% efficiency at 450 nm according to data sheet values.^36^ In addition, the sensing area of the KAF-8300 sensor is 243 mm^2^, while the sensing area of LuminoSiPM is only 1.69 mm^2^.

As concerns results obtained with the OnePlus6 CMOS-integrated sensors, LODs of 1.3 × 10^–6^ M (6.5 × 10^–12^ moles) and of 2.5 × 10^–8^ M (corresponding to 1.3 × 10^–13^ moles) were obtained for PpyGR-TS and NanoLuc, respectively (Figure 2B, D). These values were one and three orders of magnitude higher, respectively, than the LODs obtained with the LuminoSiPM. As concerns the linear range, LuminoSiPM was able to maintain a linear correlation between concentration and photon counts in the concentration range of 4.2 × 10^–7^ to 1.3× 10^–5^ M for PpyGR-TS (*R*^2^ = 0.8666) and for NanoLuc of 3.6× 10^–9^ to 5.0 × 10^–7^ M (*R*^2^ = 0.8887) (Figure 2A, C). More details are shown in Table S1.

According to a recent work, most smartphone-based sensing platforms reported in the literature are not optimized due to suboptimal design and intrinsic limitations of smartphones with very small lens apertures.^21^ Because smartphone-integrated lenses remain the factor that plays a major role in determining the final sensitivity of the sensing platform, alternative systems such as SiPM could represent a more suitable alternative to improve the assay sensitivity while keeping cost contained. Another consideration is related to the fact that most 3D-printed prototypes that have been reported by us and others fit only one model of smartphone. This reduces the general applicability of the sensors and surely hampers their market entrance. Instead, more sensitive yet cheap platforms that could be easily connected to a smartphone used only for data handling could be more advantageous. In the current configuration, the electric power was supplied by the PC USB port, but a smartphone or a cheap microcontroller display unit could be deployed for data storage and/or elaboration in perspective of having a standalone portable battery powered device with direct cloud connection. The use of the smartphone/tablet option is under development using USB CDC instead of the RS232 interface. The USB OTG port of the LuminoSiPM micro can theoretically reach 480 Mbit/s data output, thus allowing management of a higher data rate.

### LuminoSiPM Detection of Acetylcholinesterase Inhibitors with a Chemiluminescent Origami Sensing Paper

**Figure 3:**
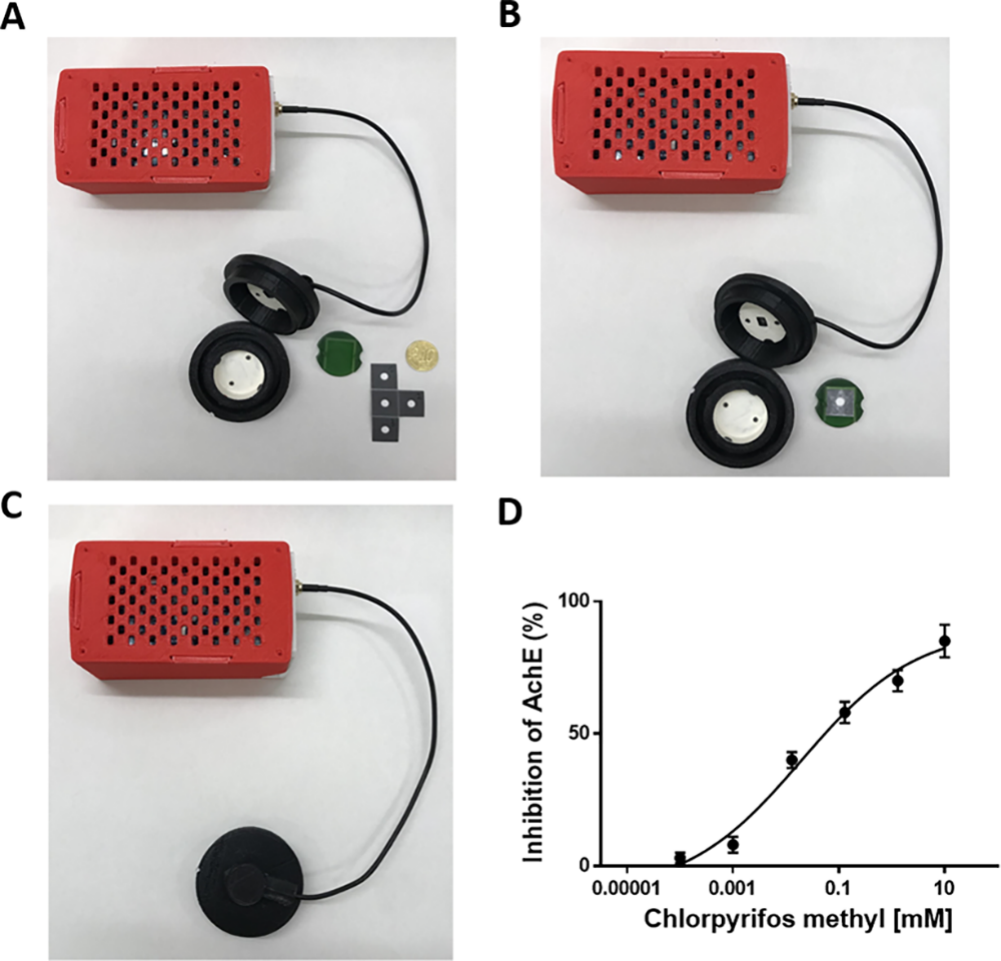
(A) Picture of the LuminoSiPM with the unfolded paper sensor, (B) the folded sensor, and (C) the assembled device. (D) Chlorpyrifos-methyl inhibition curve.

The suitability of LuminoSiPM for the detection of CL signals was also evaluated. An origami sensing paper based on the inhibition of AChE activity by molecules such as OP pesticides and nerve agents^23^ was optimized and adapted to fit the LuminoSiPM device (Figure 3).

The origami sensing paper is based on enzyme inhibition with three enzymatic reactions relying on the luminol/H_2_O_2_/HRP system. This sensing paper allows the measurement of AChE inhibitory activity via coupled enzymatic reactions in very short times (less than 30 min) using small volumes of samples (10 μL) and a very straightforward procedure (Figure S5).

A 3D printed case was fabricated to host the sensing paper and keep it folded after adding the reagents (Supporting Information). We used chlorpyrifos-methyl as the model analyte, commercially sold as insecticide Reldan 22. The calculated LOD for chlorpyrifos methyl was 0.4 μM. The use of LuminoSiPM enabled significant improvement of the LOD, corresponding to 45 μM, previously obtained exploiting CMOS-smartphone integrated detector.^23^ This proof-of-principle assay demonstrated that LuminoSiPM can be used for quantitative and rapid detection of pesticides acting on Ach and can be easily adapted to different analytical formats.

## Conclusion

In this work, we exploited SiPM technology to develop a low-cost device suitable for measuring enzyme-catalyzed biochemiluminescent reactions. In view of its application for detecting low light intensities in critical fields such as environmental and forensic analysis, the performance of the device was evaluated and compared to more explored portable light detectors such as smartphone-integrated CMOS and a portable CCD camera. A remarkable improvement of the LOD was obtained for both luciferin-luciferase reactions and in a proof-of-principle application based on a paper sensor for detecting Ach inhibitors. An impressive LOD in the femtomolar range was reported for NanoLuc with a linearity of response extending up to three orders of magnitude, thus confirming the suitability of LuminoSiPM for on-site applications. In addition, due to the temperature dependence of detector noise figures, we envisage that the implementation of temperature stabilization systems based on Peltier elements might further improve the sensitivity.

Our results support the use of the LuminoSiPM for on-site analysis, which also benefits from a relatively small footprint and low power demand.

## Supporting Information Available

Additional experimental details, figures (SiPM dark noise acquisitions, emission kinetics, and calibration curves of NanoLuc and PpyGR-TS, foldable origami sensing paper biosensor and assay procedure), and tables (LOD and linear range with the different light detectors, RSD of chlorpyrifos methyl inhibition curve) (PDF)
