# Supplementary material for: Ultrasensitive On-Field Luminescence Detection Using a Low-Cost Silicon Photomultiplier Device
Source: Anal Chem. 2021 May 11;93(20):7388–93. doi: 10.1021/acs.analchem.1c00899 (PMC8253476; doi:10.1021/acs.analchem.1c00899)
Supplement: Supplementary file 1 — ac1c00899_si_001.pdf [file ac1c00899_si_001.pdf]

SUPPORTING INFORMATION

Ultrasensitive on-field luminescence detection using a low-cost silicon photomultiplier (SiPM) device

Maria Maddalena Calabretta[1,2¥], Laura Montali[1,2¥], Antonia Lopreside[1,2], Fabio Fragapane[3], Francesco Iacoangeli[4], Aldo Roda[1,5], Valerio Bocci[4], Marcello D'Elia*[3], Elisa Michelini*[1,2,5,6]

[1]Department of Chemistry "Giacomo Ciamician", University of Bologna, 40126 Bologna, Italy

[2]Center for Applied Biomedical Research (CRBA), University of Bologna, Italy

[3]Gabinetto Regionale di Polizia Scientifica dell'Emilia-Romagna, Bologna, 40123, Italy

[4]INFN, Istituto Nazionale di Fisica Nucleare, Sezione di Roma, 00185 Rome, Italy

[5]INBB, Istituto Nazionale di Biostrutture e Biosistemi, 00136 Rome, Italy

[6]Health Sciences and Technologies-Interdepartmental Center for Industrial Research (HST-ICIR), University of Bologna, Bologna, Italy

Table of contents

Experimental section

Reagents…………………………………………………………………………………..………...3

LuminoSiPM fabrication……..……………………...…………………...……………...……….…...………3

3D printed LuminoSiPM dark box..……………….………...………………………………...……….……4

Preliminary dark count rate evaluation………...………………...……….……………………………………4

Protein purification.……...……………………….…………….……………..…………………..…...4

Evaluation of LuminoSipM analytical performance in detecting bioluminescence and comparison with other portable light detectors and benchtop instrumentation. ……………….………………………………………5

Preparation of the ready-to-use origami sensing paper. …………….…….………………………………5

LuminoSiPM detection of AchE inhibitors with a CL origami sensing paper.……………………….…….6

Figure S1. The three components of LuminoSiPM dark box……….………………………………....8

Figure S2. Series of SiPM Dark Noise acquisitions………………..……………………………….…....8

Figure S3. (A) Normalized emission spectra of NanoLuc and PpyGR-TS luciferases with the photon detection efficiency of the Hamamatsu MPPC 13360-1325CS SiPM Sensor and (B) emission kinetics of NanoLuc and PpyGR-TS…………………...………………………………………………...…………………………9

Figure S4. Calibration curves of PpyGR-TS (A) and Nanoluc (B) luciferases obtained with the Varioskan Flash multimode reader………………………………………………………..……………………...10

Figure S5. Schematic representation of the foldable origami sensing paper biosensor and assay procedure. ………………….………………………………………………………………………………..10

Table S1. Limit of detection (LODs) and linear range obtained for PpyGR-TS and Nanoluc luciferase with LuminoSiPM, ATIK 383L and OnePlus6 smartphone. ……………………………….…….………….…11

Table S2. Relative standard deviation (RSD) of Chlorpyrifos methyl inhibition curve obtained with LuminoSiPM device…………………………………………………………………………...…...11

References……………………………………………………………………………………………12

EXPERIMENTAL SECTION

Reagents

E. coli competent cells (JM109) for plasmid propagation and SOC medium (tryptone 20g/L, yeast extract 5 g/L, NaCl 5M 2 mL/L, KCl 1.0 M 2,5 mL/L, MgCl2 1.0 M 10mL/L, MgSO4 1.0 M 1 0 mL/L, D-Glucose 1.0 M 20 mL/L) were from Sigma (St. Louis, MO, USA), E. coli competent cells (BL21) for protein expression were from Agilent Technologies (Santa Clara, USA). Luria-Bertani (LB) medium and LB-Agar plates used for cell cultures were prepared with Select Agar and LB (Lennox L Broth) from Sigma (St. Louis, MO, USA) added with ampicillin (1 μg/mL). All media and materials were autoclaved for 20 minutes at 121°C. Expression plasmids, kits for plasmid extraction and purification, BrightGlo and NanoGlo substrates were from Promega (Madison, WI, USA). Enzymes required for cloning were from Fermentas (Vilnius, Lithuania). B-PER™ Bacterial Protein Extraction Reagent was from Thermo Scientific (Rockford, USA). Protino Ni-IDA Resin and 14 ml Protino Columns required for protein extraction were purchased from MACHEREY-NAGEL GmbH & Co. KG (Düren, Germany). Microcon 30K device used for protein concentration and buffer exchange was purchased from Merck Millipore Ltd. (Tullagreen, Carrigtwohill, Co. Cork, IRL). Acetylcholinesterase (AChE) from Electrophorus electricus (EC 3.1.1.7), choline oxidase (ChOx) from Alcaligenes sp. (EC 1.1.3.17), peroxidase (HRP) Type VI-A from horseradish (EC 1.11.1.7), acetylcholine (ACh) chloride, luminol sodium salt and all other chemicals were purchased from Sigma (St. Louis, MO, USA).

LuminoSiPM fabrication. A previously developed ArduSiPM board, the first all-in-one system for SiPM in literature, was selected as a compact data acquisition system for SiPM sensor with low-power consumption. ArduSiPM uses SAM3X8E 32-bit ARM® Cortex-M3 CPU as processor unit,[1] an open hardware/software development board, that contains all circuits to acquire analog and digital signals and, if programmed with suitable firmware, can output processed data.2-4 The detector can measure the relative time of occurrence of each photon burst, the number of occurrences within a specified acquisition window (one second as default), and the intensity of each blinking with single photon precision. The Hamamatsu MPPC 13360-1325CS SiPM Sensor was used. This sensor contains a sensitive matrix of 2668 GM-APD cells with a pixel pitch of 25 μm, an active photosensitive area of 1.3x1.3 mm and a fill factor of 47%. The sensor is protected by a silicon resin window and has a spectral response in the visible region, with a declared maximum photon detection efficiency (PDE) at 450 nm of 25%. The typical driving voltage is around of 50-60 V. The operative

voltage (Vop), defined as Vbreak down + 5V, is 56.45 V, with a leakage current "Id" (the intrinsic current which flows when the sensor is in the dark) of 0.013 μA at 25 °C.

3D printed LuminoSiPM dark box. The two parts of LuminoSiPM dark box were fabricated with a 3D printer (3DiELLE - ver. L) using thermoplastic black polylactic acid (PLA) polymer to shield from environmental light both sensor and sample holder. Sample-holder cartridges, designed in different versions according to the analysis format, were printed in PLA and in UV 405 nm sensitive resin, using an Anycubic Photon 3D printer (Anycubic Shenzhen, China). For measurements with liquid samples, a central cylindrical well was designed (radius 1.75 mm, height 3.0 mm - inner volume about 30 μL) (Figure S1). A sample-holder with an inner, square cavity (19x19 mm) was 3D printed in UV sensitive resin, to accommodate the foldable paper disposable device.

The sample holder was fabricated with stereolithography 3D printing, providing, when compared to components printed with PLA, better resolution and reduced surface porosity,[5] thus preventing liquid leakage. The complete polymeric composition insulated the sensor contacts operating at a static voltage of around 56V.To simplify the use of the device and reduce issues related to incorrect positioning of removable sample holders in the box, specific reference points were included with custom mechanical protrusions (pins height 5.5 mm, diameter. 1.15 mm) and complementary alignment holes in the box parts and in sample holders (Figure S1). This ensured that, when the LuminoSiPM dark box was operative, samples were always placed in the same position, in front of the detector, at about 1.5 mm from the sensitive surface.

Preliminary dark count rate evaluation. To select the most suitable instrumental condition, especially regarding the dark (thermal) noise, two series of 5 min signals acquisitions were performed, with the sensor shielded from light and varying gradually the discriminator threshold admittance in the range 3.39 - 4.57 mV. Measures were repeated at two different temperature conditions: series "A" between 24-26 °C, Series "B" between 26-28 °C (Figure S2).

Protein purification. To investigate the suitability of LuminoSipM to detect low light intensities of BL reactions, two luciferases PpyGR-TS and NanoLuc were purified as previously described.[6] Briefly, a 6-histidine sequence and a spacer (GCGHHHHHH) were fused with the C-terminal of NanoLuc and PpyGR-TS luciferases via standard Polymerase Chain Reaction (PCR) technique. PCR products were subsequently inserted into pGex-6P-2 plasmid using BamHI and NotI for high protein expression in *E. coli* strain BL21. Protein purification was performed using whole cell extracts after 4 h incubation with isopropyl β-D-1-thiogalactopyranoside (IPTG) 0.1 mM at 25°C, with Protino Ni-

IDA Resin according to the manufacturer's instructions. Cell extract was obtained using a cell-lysis-extraction buffer consisted of B-PER™ Reagent (from Thermo Scientific, Rockford, USA), Lysozyme (0.1 mg/mL) and phenylmethylsulfonyl fluoride (10 µM), incubated for 20 min, on ice. Proteins were purified using Protino Ni-IDA Resin and LEW Buffer as solution. LEW Buffer pH 8.0 was prepared using NaCl 300 mM and NaH_2PO_4 50 mM. LEW Buffer plus Imidazole 250 mM and glycerol 2% was used for protein elution and storages (at 4 °C). Protein concentrations were determined with the Bradford Reagent from Sigma (St. Louis, MO, USA) using bovine serum albumin (BSA) as standard. Protein purification yields were ~5 mg/0.25 L culture for both the luciferases.

Evaluation of LuminoSipM analytical performance in detecting bioluminescence and comparison with other portable light detectors and benchtop instrumentation. The sensitivity, dynamic range, and linearity of the system response were assessed using luciferin-luciferase solutions containing all substrates and cofactors required for the firefly luciferase BL reaction:

$$Luc + LH_2 + ATP \overset{Mg^{2+}}{\leftrightarrows} Luc \cdot LH_2 - AMP + Ppi \quad (1)$$

$$Luc \cdot LH_2 - AMP + O_2 \rightarrow Luc \cdot Oxyluciferin^* + AMP + CO_2 \quad (2)$$

$$Luc \cdot Oxyluciferin^* \rightarrow Luc \cdot Oxyluciferin + hv \quad (3)$$

and for the NanoLuc-catalyzed BL reaction:

$$NanoLuc + Furimazine + O_2 \rightarrow Furimamide + CO_2 + hv \quad (4)$$

BL measurements obtained with ATIK 383L+ monochrome CCD were performed integrating BL signals for 5 min at +25°C (room temperature). For BL measurements performed with Oneplus 6 smartphone, images were taken for 30 sec with different sensitivity settings, from ISO 100 to ISO 3200. Images were elaborated with ImageJ software (National Institutes of Health, Bethesda, MD) to quantify the signal over the sample spot area and expressed as relative light units (RLUs). GraphPad Prism v.5 software (GraphPad Software, La Jolla, USA) was used to analyze data. Limit of Detection (LOD) was calculated as the blank signal plus three times the standard deviation. All measurements were performed in triplicate for each concentration tested and repeated at least three times with the same disposable sample-holder cartridge used for LuminoSiPM. A standard benchtop luminometer Varioskan Flash multimode reader (ThermoFisher Scientific) was used for characterization of the purified PpyGR-TS and Nanoluc luciferases. BL emission spectra (350–700 nm) and kinetics (30 min, 300 ms integration time) were obtained with a black 384-well microtiter plate after the addition of a

5 μL-volume of 0.1 mg/mL purified protein solution and 10 μL of BL substrate (BrightGlo substrate for PpyGR-TS and NanoGLo for NanoLuc) (Figure S3). Calibration curves of PpyGR-TS and NanoLuc luciferases were also obtained with the Varioskan Flash multimode reader (Figure S4).

Preparation of the ready-to-use origami sensing paper. The origami sensing paper, composed by Whatman 1 CHR cellulose chromatography paper from GE Healthcare (Chicago, IL, USA) was designed using PowerPoint (Microsoft, Redmond, WA, USA) and printed using a ColorQube 8570 office wax printer (Xerox, Norwalk, CT, USA). The origami sensing paper consisted of four circular hydrophilic "wells" (diameter 5 mm), numbered from 1 to 4 according to the folding sequence, surrounded by hydrophobic areas. The internal 3D-wax structure confining wells was obtained through a heating process for 2 min at 84 °C. Acetylcholinesterase (AChE) from *Electrophorus electricus*, choline oxidase (ChOx) from *Alcaligenes sp.*, peroxidase (HRP) Type VI-A from horseradish were then loaded in the origami sensing paper by dispensing appropriate volumes of enzyme solutions in the wells (5 μL of a 100 U/mL AChE solution in well No.1,15μL of a 20U/mL ChOx solution in well No.2, and 15 μL of a 108 U/mL HRP solution in well No. 3). Finally, the origami sensing paper was let drying for 30 min at 37°C. A 0.025 M luminol solution in NaOH at pH 12.0 was used as substrate for the chemiluminescent reaction.

LuminoSiPM detection of AchE inhibitors with a CL origami sensing paper. The analytical procedure consists in addition of a 10 μL-volume of sample to well No. 1 (which contains AChE) and pre-incubation at room temperature for 5 minutes. After folding of well No. 1 on the central well, a 10 μL-volume of Ach solution (10 mM) is added and Ach hydrolysis occurs. Then, well No. 2 (which contains ChOx) is folded on the central well leading to production of choline and then to hydrogen peroxide. After 5-min incubation, well No. 3, containing HRP, is folded on the central well to obtain the completely folded paper. The biosensor is clamped into the sample holder (Figure S5) and a 20 μL-volume of 0.025 M luminol in NaOH (pH 12.0) is added to trigger HRP-catalyzed CL reaction leading to the production of CL signal. The sample holder is then inserted in the LuminoSiPM dark box and then, after 10 min of incubation, closed with the other section of the dark box (which supports the sensor) to acquire the CL signal.

For all experiments, two separate measurements were performed with the same LuminoSiPM settings described in previous section to assess AChE inhibition either in the presence or in the absence of the inhibitor (in this case 10 μL of deionized water were used in the pre-incubation step). All measurements were performed in triplicate and repeated at least three times. Calibration curves for chlorpyrifos-methyl were obtained by analyzing with the origami sensor and LuminoSiPM device

chlorpyrifos methyl solutions in the concentration range 1.0 x 10^{-4} – 10.0 mM and fitting the dose/inhibition graph with a four-parameter logistic equation.

The optimized analytical procedure to evaluate the activity of AChE inhibitors in a liquid sample is shown in Figure S5. A 10 μL-volume spiked Reldan®22 solution in water corresponding to chlorpyrifos methyl (concentrations range 0.0001 – 10.0 mM) was added to the well No. 1 of the origami sensing paper (which contains the AChE enzyme) and let to incubated for 5 minutes at room temperature so that inhibition of AChE takes place (Figure S3A). After folding of well No. 1 on the central well (Figure S3B) and addition of 10 μL of ACh solution (10 mM in deionized water) the AChE-catalyzed hydrolysis of ACh takes place. Then, the well No. 2 (which contains ChOx) is folded on the central well to activate the enzyme reactions leading to choline and then to hydrogen peroxide, and the partially folded origami sensing paper is clamped by the 3D-printed sample-holder cartridge (Figure S3C). After a second 5-min incubation, the well No. 3 containing HRP is folded on under the already stacked wells to obtain the completely folded origami sensing paper (Figure S3D-E). After clamping the biosensor in the holder again, 20 μL of a 0.025 M luminol solution in NaOH (pH 12.0) are added to trigger the final HRP-catalyzed CL reaction leading the production of the CL signal (Figure S3F). The sample-holder cartridge is then inserted in the LuminoSiPM dark box and after 10 min of incubation, closed with the other section of the dark box (which supports the sensor) to acquire the CL signal for 5 min.

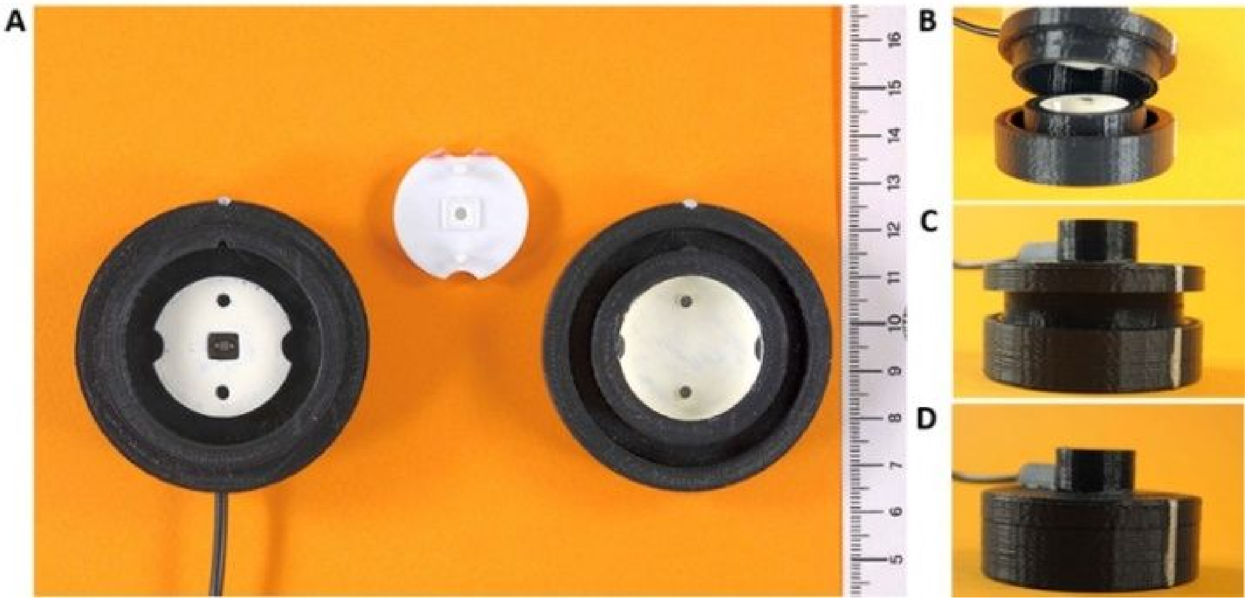

Figure S1. (A) The three components of LuminoSiPM dark box. (B) The assembly of sample holder in the dark box. (C) Mechanical protrusions and complementary alignment holes guarantee light tightness. (D) The box closed and ready for measurement.

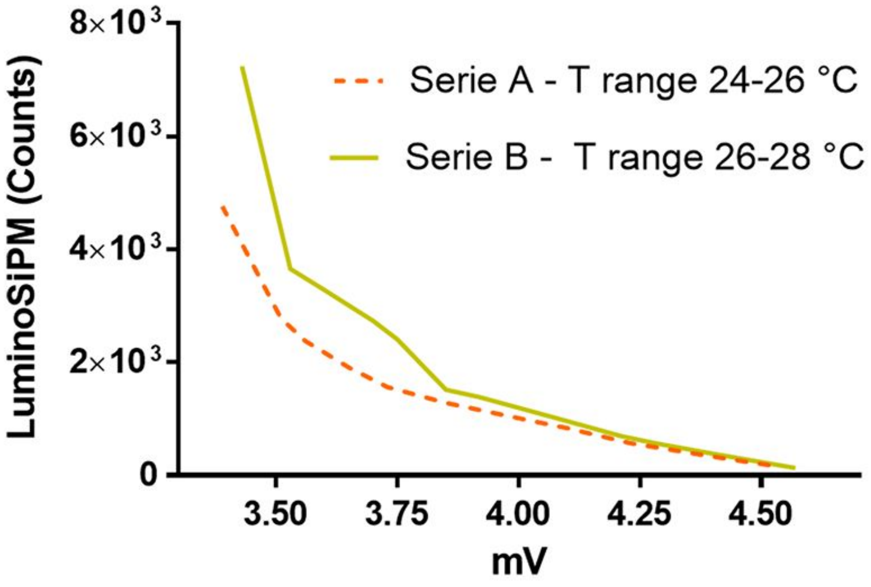

Figure S2. Series of SiPM Dark Noise acquisitions: Series A measurements acquired at room temperature range 24-26 °C (dotted line); Series B measurements acquired at room temperature range 26-28 °C (solid line).

A

B

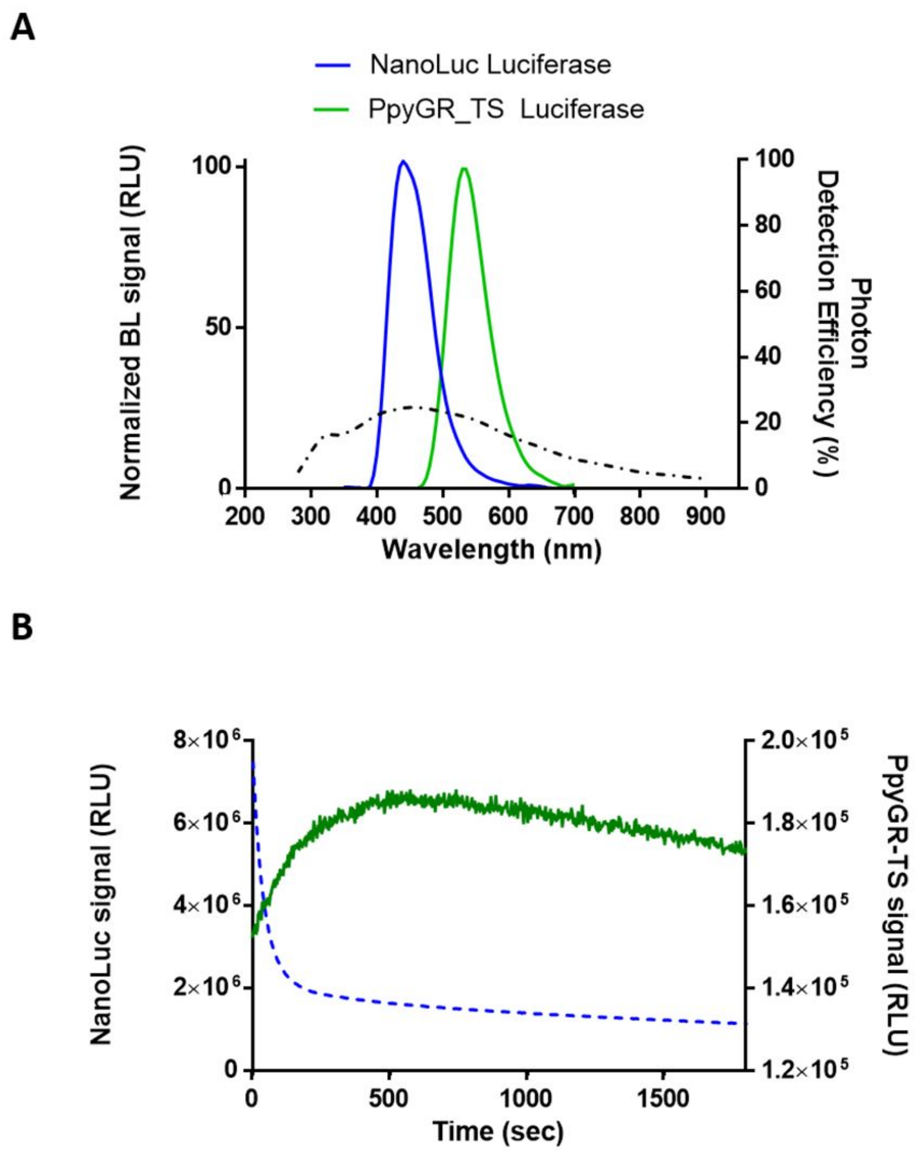

Figure S3. (A) Normalized emission spectra of NanoLuc and PpyGR-TS luciferases with the photon detection efficiency of the Hamamatsu MPPC 13360-1325CS SiPM Sensor (dashdotted line); (B) Emission kinetics of NanoLuc (dashed blue line) and PpyGR-TS (solid green line).

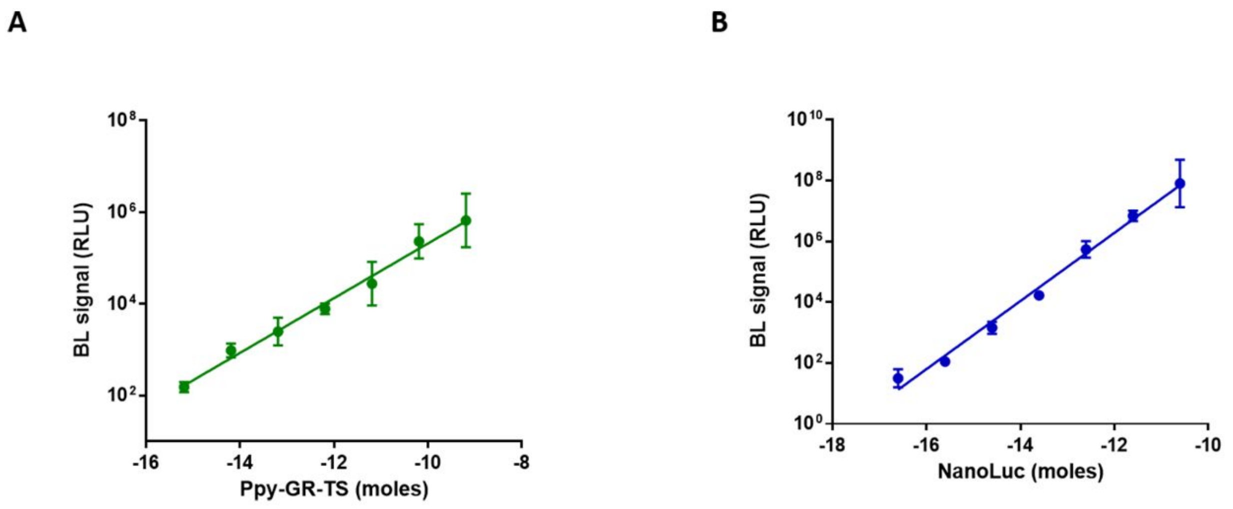

Figure S4. Calibration curves of PpyGR-TS (A) and Nanoluc (B) luciferases obtained with the Varioskan Flash multimode reader.

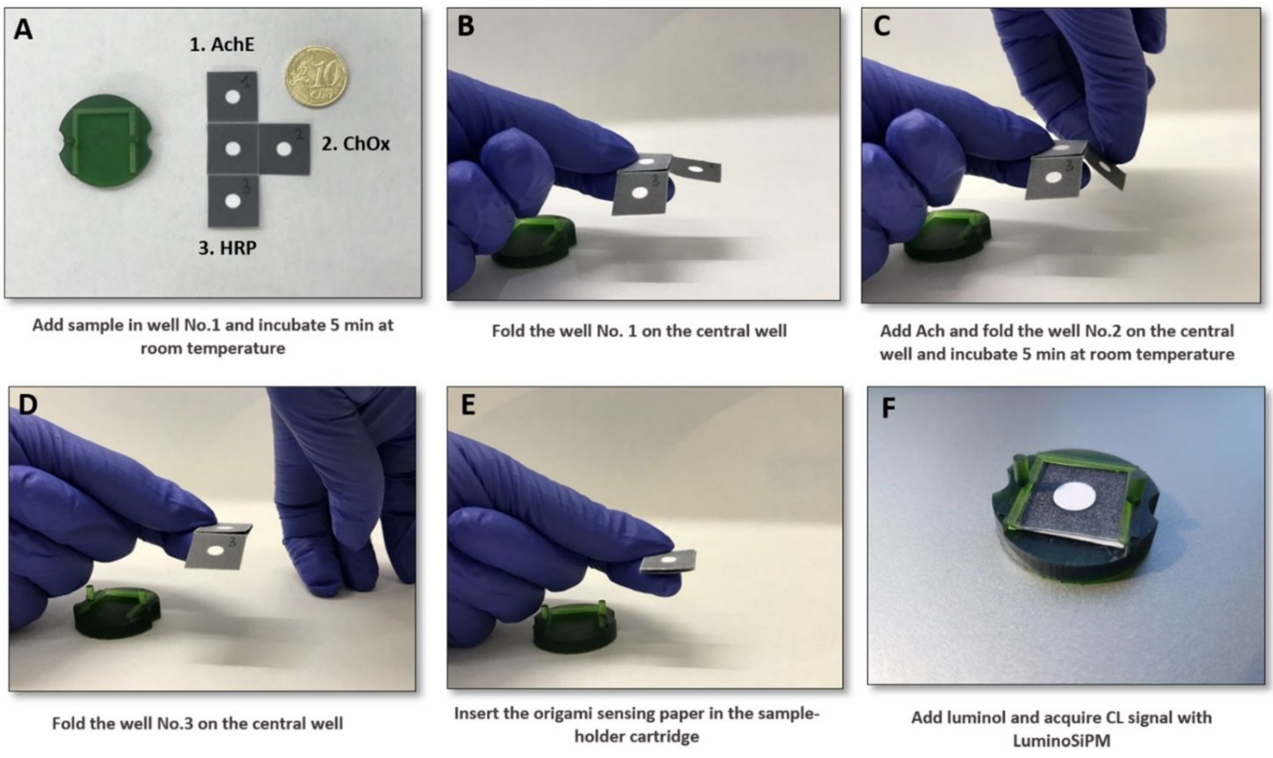

Figure S5. Schematic representation of the foldable origami sensing paper biosensor and assay procedure.

Table S1: Limit of detection (LODs) and linear range obtained for PpyGR-TS and Nanoluc luciferase with LuminoSiPM, ATIK 383L and OnePlus6 smartphone.

	**PpyGR-TS LOD**	**NanoLuc LOD**	**PpyGR-TS LINEAR RANGE**	**NanoLuc LINEAR RANGE**
**LuminoSiPM**	2.1×10^{-13} moles (4.2×10^{-8} M)	8.7×10^{-16} moles (1.7×10^{-10} M)	2.1×10^{-12} – 6.5×10^{-11} moles (4.2×10^{-7} – 1.3×10^{-5} M)	1.8×10^{-14} – 2.5×10^{-12} moles (3.6×10^{-9} - 5.0×10^{-7}M)
**ATIK 383L+**	7.0×10^{-13} moles (1.4×10^{-7} M)	2.2×10^{-14} moles (4.3×10^{-9} M)	3.0×10^{-12} – 6.5×10^{-11} moles (6.0×10^{-7} – 1.3×10^{-5} M)	3.8×10^{-14} – 2.5×10^{-11} moles (5.0×10^{-6} – 7.6×10^{-9} M)
**OnePlus6 Smartphone**	6.5×10^{-12} moles (1.3×10^{-6} M)	1.3×10^{-13} moles (2.5×10^{-8} M)	n.d.	6.5×10^{-12} - 2.5×10^{-10} moles (1.3×10^{-6} – 5.0×10^{-5} M)

Table S2: Relative standard deviation (RSD) of Chlorpyrifos methyl inhibition curve obtained with LuminoSiPM device

**Chlorpyrifos methyl [mM]**	**RSD (%)**
0.0001	62
0.001	36
0.013	8
0.13	7
1.3	6
10.0	7

References

(1) Kim, S.B.; Paulmurugan, R. Bioluminescent Imaging Systems for Assay Developments. *Anal Sci.* **2020**, 20R003.

(2) Collamati, F.; Bocci, V.; Castellucci, P.; De Simoni, M.; Fanti, S.; Faccini, R.; Giordano, A.; Maccora, D.; Mancini-Terracciano, C.; Marafini, M.; Mirabelli, R.; Morganti, S.; Schiavina, R.; Scotognella, T.; Traini, G.; Solfaroli Camillocci, E. Radioguided surgery with β radiation: a novel application with Ga68. *Sci Rep.* **2018**, *8*, 1-9.

(3) Carlotti, D.; Collamati, F.; Faccini, R.; Fresch, P.; Iacoangeli, F.; Mancini-Terracciano, C.; Marafini, M.; Mirabelli, R.; Recchia, L.; Russomando, A.; Solfaroli Camillocci, E.; Toppi, M.; Traini, G.; Bocci, V. Use of bremsstrahlung radiation to identify hidden weak β− sources: feasibility and possible use in radio-guided surgery. *JINST* **2017**, *12*, P11006–P11006.

(4) Scandale, W.; Andrisani, F.; Arduini, G.; Cerutti, F.; Garattini, M.; Gilardoni, S.; Masi, A.; Mirarchi, D.; Montesano, S.; Petrucci, S.; Redaelli, S.; Schoofs, P.; Rossi, R.; Breton, D.; Chaumat, D.; Dubos, S.; Maalmi, J.; Natochii, A.; Puill, V.; Raymond, M. Study of inelastic nuclear interactions of 400 GeV/c protons in bent silicon crystals for beam steering purposes. *Eur. Phys. J. C* **2018**, *78*, 1-8

(5) Al-Maharma, A. Y.; Patil, S.P.; Markert, B. Effects of porosity on the mechanical properties of additively manufactured components: a critical review. *Mat. Res. Express* **2020**, *7*, 12200

(6) Branchini, B. R.; Ablamsky, D. M.; Murtiashaw, M. H.; Uzasci, L.; Fraga, H.; Southworth, T. L. Thermostable red and green light-producing firefly luciferase mutants for bioluminescent reporter applications. *Anal. Biochem.* **2007**, *361*, 253–262.
